# Impact of COVID-19 on the mental health of US college students

**DOI:** 10.1186/s40359-021-00598-3

**Published:** 2021-06-08

**Authors:** Jenny Lee, Matthew Solomon, Tej Stead, Bryan Kwon, Latha Ganti

**Affiliations:** 1 Brown University, Providence, RI USA; 2 Envision Physician Services, Plantation, FL USA; 3 Departments of Emergency Medicine and Neurology, University of Central Florida, 6950 Lake Nona Blvd, Orlando, FL 32832 USA; 4 Ocala Regional Medical Center, Ocala, FL USA

**Keywords:** COVID-19, Mental health, Pandemic, Anxiety, Depression, Loneliness

## Abstract

**Background/aim:**

In the beginning of 2020, the novel Coronavirus disease (COVID-19) caused by the SARS-CoV-2 virus, became a public health emergency in the U.S. and rapidly escalated into a global pandemic. Because the SARS-CoV-2 virus is highly contagious, physical distancing was enforced and indoor public spaces, including schools and educational institutions, were abruptly closed and evacuated to ensure civilian safety. Accordingly, educational institutions rapidly transitioned to remote learning. We investigated the impacts of the COVID-19 pandemic on domestic U.S. college students, ages 18–24 years.

**Methods:**

Through Pollfish®’s survey research platform, we collected data from 200 domestic U.S. college students in this age range (N = 200) regarding the physical, emotional, and social impacts of COVID-19 as well as key background information (e.g. whether or not they are first-generation or if they identify with the LGBTQIA+ community).

**Results:**

Our results indicate that students closer to graduating faced increases in anxiety (60.8%), feeling of loneliness (54.1%), and depression (59.8%). Many reported worries for the health of loved ones most impacted their mental health status (20.0%), and the need to take care of family most affected current and future plans (31.8%). Almost one-half of students took to exercising and physical activity to take care of their mental health (46.7%). While a third did not have strained familial relationships (36.5%), almost one half did (45.7%). A majority found it harder to complete the semester at home (60.9%), especially among those who had strained relationships with family (34.1%). Seventy percent spent time during the pandemic watching television shows or movies. Significantly more men, first-generation, and low-income students gained beneficial opportunities in light of the pandemic, whereas their counterparts reported no impact. First-generation students were more likely to take a gap year or time off from school.

**Conclusions:**

Although students found ways to take care of themselves and spent more time at home, the clear negative mental health impacts call for schools and federal regulations to accommodate, support, and make mental health care accessible to all students.

**Supplementary Information:**

The online version contains supplementary material available at 10.1186/s40359-021-00598-3.

## Introduction

The outbreak of the SARS-CoV-2 virus, originally from Wuhan, China, in December 2019 gave rise to an ongoing global public health crisis addressing the proliferation of the novel Coronavirus disease (COVID-19). The SARS-CoV-2 virus directly targets the respiratory system in humans with characteristic symptoms of cough, fever, sore throat, dyspnea, and fatigue [1]. As the virus continued spreading over 2020, more findings on the epidemiological characteristics of SARS-CoV-2 have been divulged to the public. A study showed that the COVID-19 disease presents varying lengths of incubation periods, typically an average of 5.2 days, and it is estimated to have a slightly higher basic reproduction number (2.24–3.58) than that of the original SARS-CoV virus [1]. According to the National Health Commission of China, the virus can be transmitted through aerosols, droplets, contacts, respiratory aspirates, and feces, with both animals (e.g., bats, pangolins) and humans being veritable modes of transmission [2]. Accurate and accelerated testing is necessary to control this extremely contagious disease in cities, communities, and hospitals.

Due to the sudden outbreak of COVID-19, most universities across the United States were forced to send their students home early for the 2019–2020 academic year to prevent spread and protect students as well as surrounding communities. The sudden change in students’ learning environment, the quality of their education, and other circumstances caused students to face unique challenges, adversely impacting their mental health. The loss of internships, on-campus jobs, and other opportunities also contributed to the stress and declining mental health of students. According to a study done on a cohort of students attending Dartmouth College, there were noticeable differences in behavioral and mental health over the course of the pandemic thus far, with a higher number of self-reported cases of depression and anxiety around final exams [3].

Other minority communities also faced particular hardship in light of the pandemic. For example, greater proportions of the lesbian, gay, bisexual, transgender, and queer or questioning (LGBTQ) population do not have access to health insurance and struggle with poverty compared to the non-LGBTQ population [4]. Because of their health disparities and social disadvantages, the mental health of LGBTQ students is exacerbated due to the psychological trauma that can come with the COVID-19 pandemic. Furthermore, people of color and those in socioeconomically disadvantaged groups are more likely to be mentally overwhelmed due to the unequal burden of finances, illness, and death. In a study done at a hospital in northern California of COVID-19 patients, non-Hispanic African Americans were found to be 2.7 times more likely to be hospitalized compared to non-Hispanic white patients [5].

A study of college students from India [6] found that both anxiety and depression were prevalent in their cohort, with women being affected more. They also noted a disturbed sleeping pattern which aligns with both anxiety and depression. A study that used smartphone-based ecological momentary assessments of anxiety and optimism related to COVID-19 and other generic mental health variables 6 times daily [7] found widespread mental health impact, especially anxiety, in their cohort of 140 students. Yet another study of college students [8] found a significant decline in physical activity and mental health occurred as a result of the COVID-19 pandemic.

There are many variables that can further contribute to the mental health status of college students during the pandemic, including their identity, family life, and background. We aimed to investigate the emotional, physical and social impacts on domestic US college students ages 18–24 and determine whether these impacts were significant among particular groups.

## Methods

### Recruitment and data collection

Two hundred (N = 200) domestic U.S. college students ages 18–24 attending a 4-year university in person before the COVID-19 pandemic were surveyed through Pollfish®. Pollfish® is a survey research platform that uses organic sampling built on Random Device Engagement (RDE). [9] Using artificial intelligence (AI) to track unique respondent identification, RDE reaches users in their natural environments as they participate in their daily activities through any device. [10] Pollfish®’s partnerships with over 120,000 applications and more than 700 million global users allow for random recruitment of participants fitting the specific inclusion criteria via in-app incentives specific to each user’s real-time activity on their respective devices [9]. The advanced AI technology and algorithm prevents fraud from single users on multiple accounts (SUMA) and suspicious or illogical responses to specific questions [10]. Pollfish® uses weighting to match the univariate distributions of age, gender, and geographic region. All results reported use this weighting.

### The survey

Two screening questions were used to determine survey eligibility. These questions inquired whether participants are male or female aged 18–24 years, and whether they attended a domestic four-year U.S. college or university. The survey then consisted of 14 multiple choice questions. For some of the questions, multiple selections amongst the multiple choices were allowed, so that percentage totals could exceed 100%. The first 3 questions of the 14 inquired about background, including the participants’ year in college, whether they are the first in their family to attend college, and if they identify with the LGBTQIA+ community. The subsequent questions honed in on the physical, emotional, and social impacts of the COVID-19 pandemic. The final question was an open-ended one designed to capture the students’ verbatim feelings.

### Statistical analysis

Data were analyzed using JMP Pro 14.1 for Windows [11]. Participants with a household income less than $50,000 annually were considered “low-income”. For comparing 2 × 2 contingency tables, Fisher’s two-tailed exact test was used. For comparing ordinal data, Wilcoxon’s rank-sum test was used. Ninety-five percent confidence intervals (CI) for odds ratios are Wald-based. All results used weighting generated by Pollfish® to match the univariate distributions of age and gender.

### Ethics committee approval

This study (# 2020-966) was considered exempt by our institutional review board manager HCA Centralized Algorithms for Research Rules on IRB Exemptions (CARRIE).

## Results

A total of 200 people responded, of whom 50.6% were female (after adding weighting). Twenty three and 3/10% were first-year students, 39.9% were second-year students, 17.8% were third-year, 12.6% were fourth-year (seniors), and seven and one half percent were taking additional semesters (fifth or higher year). Fifty eight percent were first-generation students, and 36% considered themselves to be “a member of the LGBTQIA+ community.” The distributions of responses to each question are summarized in the Appendix, and each question is explored in detail in this section.

### How has COVID-19 impacted your mental health?

**Fig. 1:**
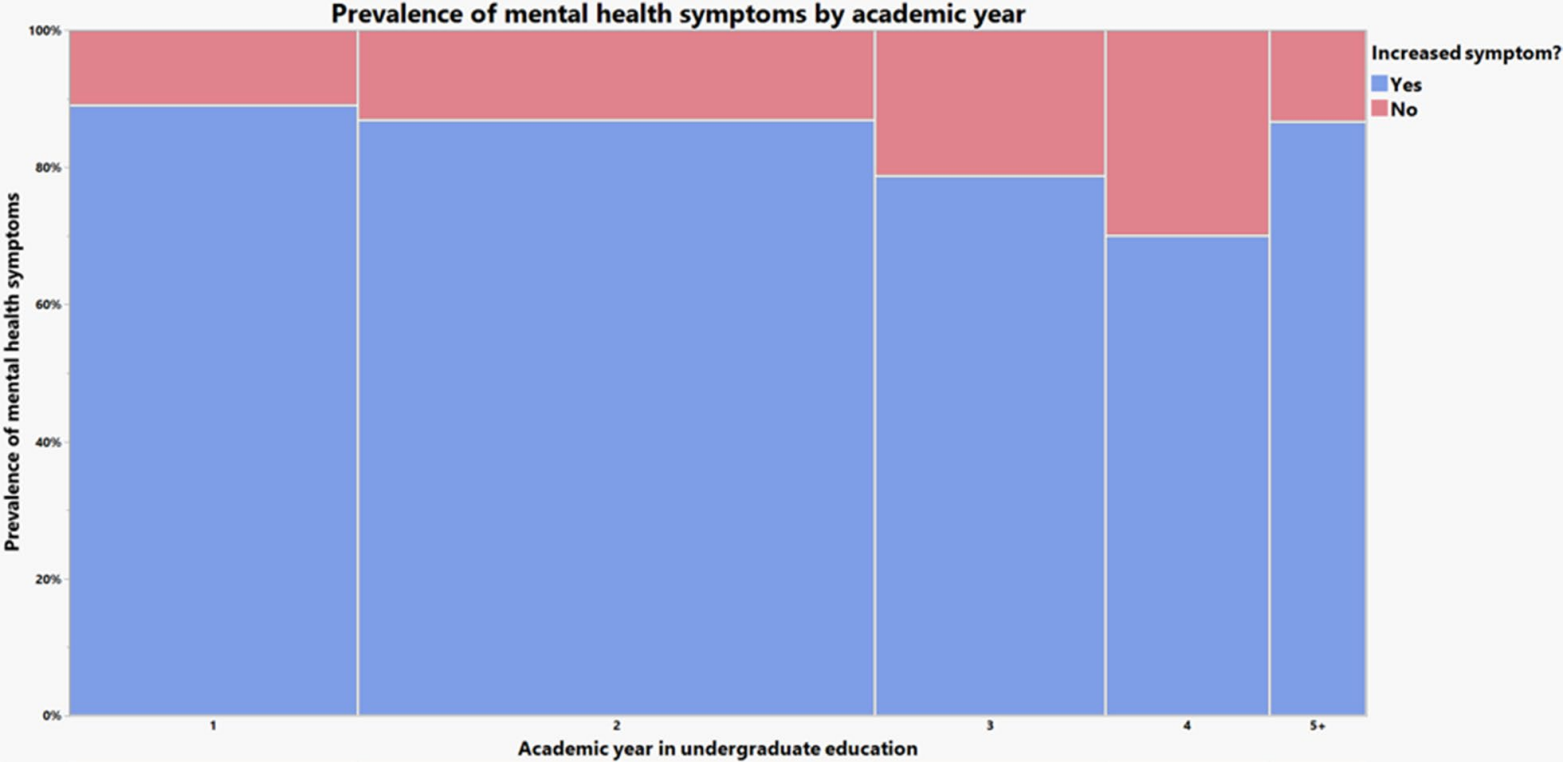
Prevalence of mental health symptoms by academic year, and whether they increased

For this question, multiple responses were allowed. Increased anxiety, depression, and feeling of loneliness were found in 60.8%, 54.1%, and 59.8% of the weighted population, respectively (Fig. 1). More than eighty percent (83.8%) reported an increase in at least one of these three symptoms. On the other hand, decreased anxiety, depression, and feelings of loneliness were respectively found in only 9.1%, 5.3%, and 4.6% of the population. For 10.7%, their mental health was unaffected. Using Fisher’s exact test, we found no significant differences in the prevalence of having at least one increased mental health symptom across first-generation status, gender, or LGBTQIA+ status. A general trend of decreased prevalence of symptoms as students drew closer to graduation was noticed. Using Fisher’s exact test, academic year and prevalence of mental health symptoms are not independent, with *p* < 0.0001.

### If you are not feeling at ease, what contributes MOST to your mental health status?

**Fig. 2:**
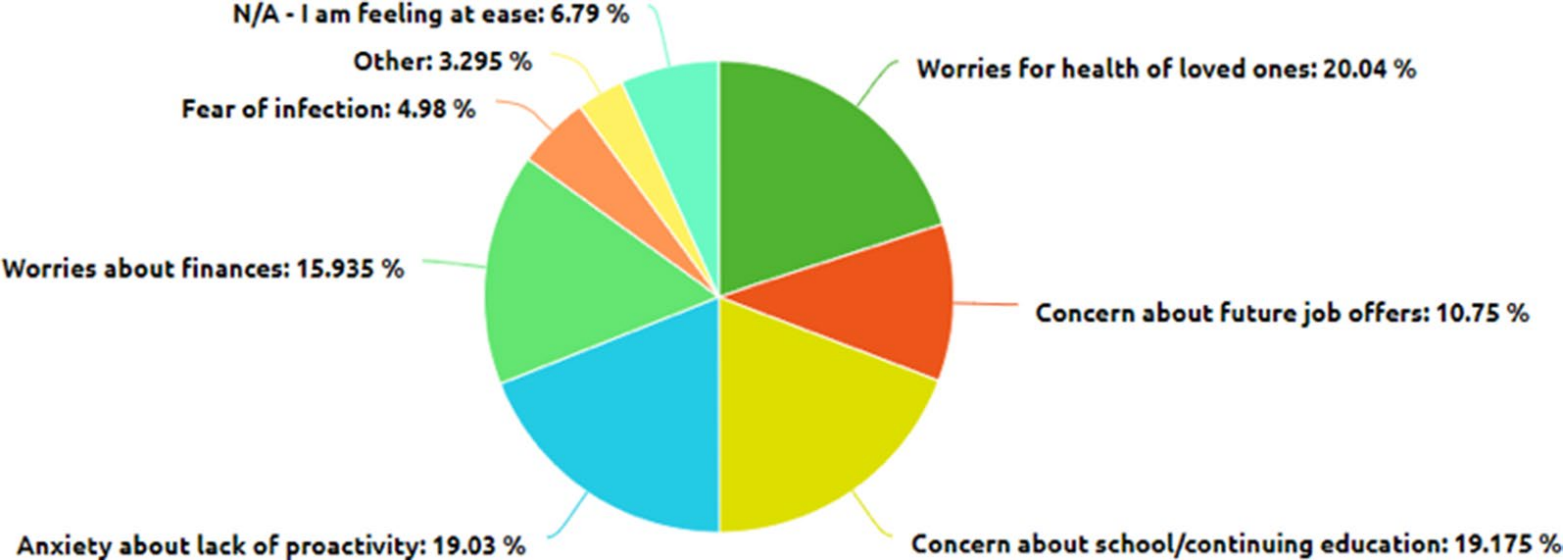
Causes of worry among students

Twenty percent of respondents said that worries about the health of loved ones was their primary concern, 19.2% were most concerned about school/continuing education, 19.0% had anxiety about lack of proactivity, 15.9% were worried about finances, 10.8% were worried about future job offers, and 5.0% feared contracting the virus. Only 6.8% said that they were feeling at ease. Interestingly, there was no significant association between the proportion of respondents who were most worried about finances and income level (Fig. 2).

### Was it easier or harder to complete this semester away from campus?

Almost sixty-one percent of students found it harder to complete the semester away from campus, while 32.7% found it easier and 6.4% reported no change. There was no significant difference across age, gender, income level, LGBTQIA+ status, or first-generation status in this response.

### How has COVID-19 affected your physical health?

Fifty percent of respondents indicated that they gained weight due to increased eating, while 20.2% reported that they felt/looked better due to exercise or dieting, 13.3% lost weight due to a lack of appetite, and 16.6% reported no changes. Among the students who reported an increased feeling of loneliness (59.8%), 57% reported that they gained weight, compared with just 39.4% of those who did not experience symptoms. Using Fisher’s exact test, this was significant with a *p*-value of 0.0301. The odds ratio (OR) is 2.04 (95% CI 1.15–3.62).

### How has COVID-19 impacted your current and future plans?

COVID-19 disrupted the lives of most students, with only 26.4% stating the pandemic has not impacted their current or future plans. 27.1% lost an internship or job offer, 22.9% are taking a gap year or time off from school, and 31.8% need to support family. Only 16.6% say that the pandemic has given them other beneficial opportunities.

The groups of people most likely to have other beneficial opportunities due to the pandemic were men (OR 3.18, 95% CI 1.41–7.22, p = 0.0039), first-generation students (OR 2.27, 95% CI 0.99–5.19, *p* = 0.0329), and low-income students (OR 3.05, 95% CI 1.35–6.99, *p* = 0.0042). First-generation students were significantly more likely to take a gap year/time off from school (OR 2.42, 95% CI 1.17–5.02, *p* = 0.0251).

On the other hand, the groups that were significantly more likely to report no impact on future plans were women (OR 2.05, 95% CI 1.07–3.92, *p* = 0.0159), non-first-generation students (OR 3.12, 95% CI 1.62–5.97, *p* = 0.0003), and non-low-income students (OR 3.77, 95% CI 1.89–7.53, *p* < 0.0001). Given that these three groups correspond exactly to those which were least likely to say they gained beneficial opportunities, we investigated the rate at which gender, first-generation status, and low-income status affects having either a beneficial opportunity or no change in future plans. When performing this analysis, we found no significant differences across any of the three groups. Combined with the other evidence, this suggests that the key difference between demographic groups lies in the rate at which they gained beneficial opportunities in light of the pandemic.

### How has COVID-19 impacted your relationships with your family?

29.4% of students had improved relationships with family, while 34.1% had strained relationships with family and 36.5% had no impact on relationships with family. The people who had strained relationships were significantly more likely to consider it harder to complete the semester at home (OR 2.59, 95% CI 1.36–4.94, *p* = 0.0036) compared to those who had improved relationships or no change.

### How has COVID-19 impacted your relationships with your friends?

27.8% of students had improved relationships with friends, while 45.7% had strained relationships with friends and 26.5% had no impact on relationships with friends.

### How have you specifically taken care of your mental health amidst COVID-19?

29.0% of students engaged in mindfulness activities (meditation, yoga, journaling, etc.). 46.7% were exercising or engaging in physical activity, 22.0% were using a health app, 17.7% were obtaining mental health care from a professional, and 30.3% were not taking any specific actions to take care of their mental health. Of the people who were exercising, 35.9% gained weight while 32.0% said they felt/looked better due to exercise or dieting. In contrast, of those who were not exercising, 62.2% gained weight and only 9.8% said they felt or looked better. Using Fisher’s exact test, this effect was significant with *p* < 0.0001.

### At what point was your concern about COVID-19 heightened?

29.8% of students had their concern first heightened when college campuses sent students home, whereas 29.0% were first alarmed by states beginning lockdown guidelines. For 10.5%, they became more concerned when a friend or relative was diagnosed with COVID-19. For just 6.4%, the turning point was that friends or relatives were taking prevention measures seriously. Only 4.1% of the population indicated that they were not concerned about COVID-19. The distribution of responses was roughly equal across demographic groups.

### How are you spending most of your time during the pandemic?

**Fig. 3:**
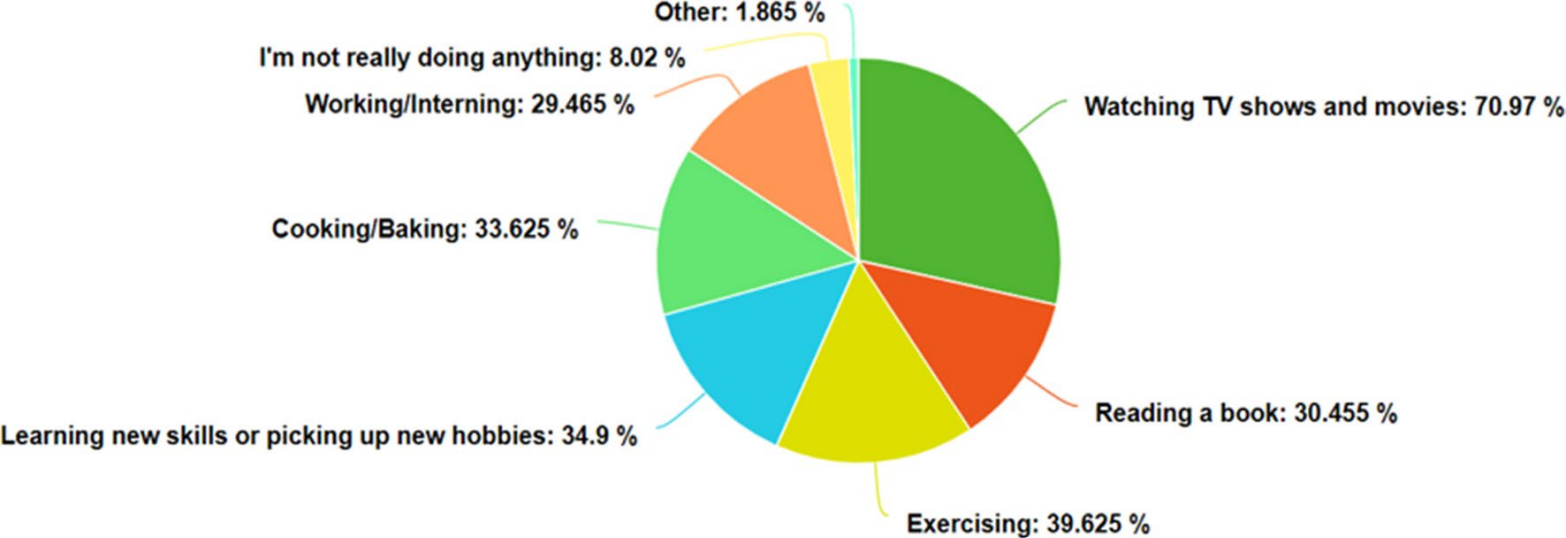
How college students spent their time during the COVID-19 pandemic

71.0% were watching TV shows or movies, 30.5% were reading books, 39.6% were exercising, 34.9% were learning new skills or picking up new hobbies, 33.6% were cooking or baking, 29.5% were working or interning, and 8.0% said they were not doing very much at all (Fig. 3). Note that the 39.6% figure of those exercising does not conflict with the 46.7% figure above, as some of the respondents may not be devoting very much time to exercise, and do not consider it a major use of their time during the pandemic.

### Is there anything else you would like to share about the effect of COVID-19 on your mental health?

As this was a free-form text box, we used JMP’s text analysis suite to identify keywords. However, neither keyword identification nor singular value decomposition provided very much insight into the sentiments expressed. Given that this was an optional question, there was not enough information for numerical patterns to emerge. Instead, we have included a few quotes that demonstrate the range of ways that the COVID-19 pandemic has affected people’s mental health (not ordered by frequency).

- “Everything has been so uncertain and has made me increasingly anxious”
- “It is comforting to know that every college student in the country, and most around the world, are dealing with the same struggles that I am, and I am not alone in being fearful and anxious.”
- “I smoke more”
- “Its very hard to hold it together knowing the world as we once knew is no longer in existence..”
- “Not getting help from my college financially has really drained me from even thinking about returning to college for FALL semester 2020.”
- “One good thing is that it's given me more time to sleep, which has been one of the only benefits of the lockdown/pandemic.”
- “It has increased productivity in approaching music & business”

## Discussion

The COVID-19 pandemic has brought severe educational and social repercussions, including the closure of college campuses as well as the introduction of online learning and social distancing at universities for the foreseeable future. College students were forced to abandon the social advantages of the so-called “college experience” (i.e. social gatherings, group studying, and in-person classes and meetings) to return home mid-semester. As reported by our survey, this triggered a decline in mental health measured by self-reported increased anxiety, depression, and loneliness. Consistent with these results, reports indicate that people of student status and ages 18–24 are at higher risk of anxiety and depression [12]. The stress associated with this abrupt social change, the disruptive factors that may exist at one’s home, and the fear of potentially contracting or spreading COVID-19 likely contributed to the development of trauma or stress-related disorders [13]. This accounts for the worsening mental health of domestic U.S. college students as shown by this survey, with significantly greater prevalence of symptoms among underclassmen that decreases with students closer to undergraduate completion. In line with the impacts we will discuss, this is likely due to the challenge of transitioning to college amidst a pandemic, the limited opportunities to form solid friendships, as well as a lack of human contact and in-person support.

**Fig. 4:**
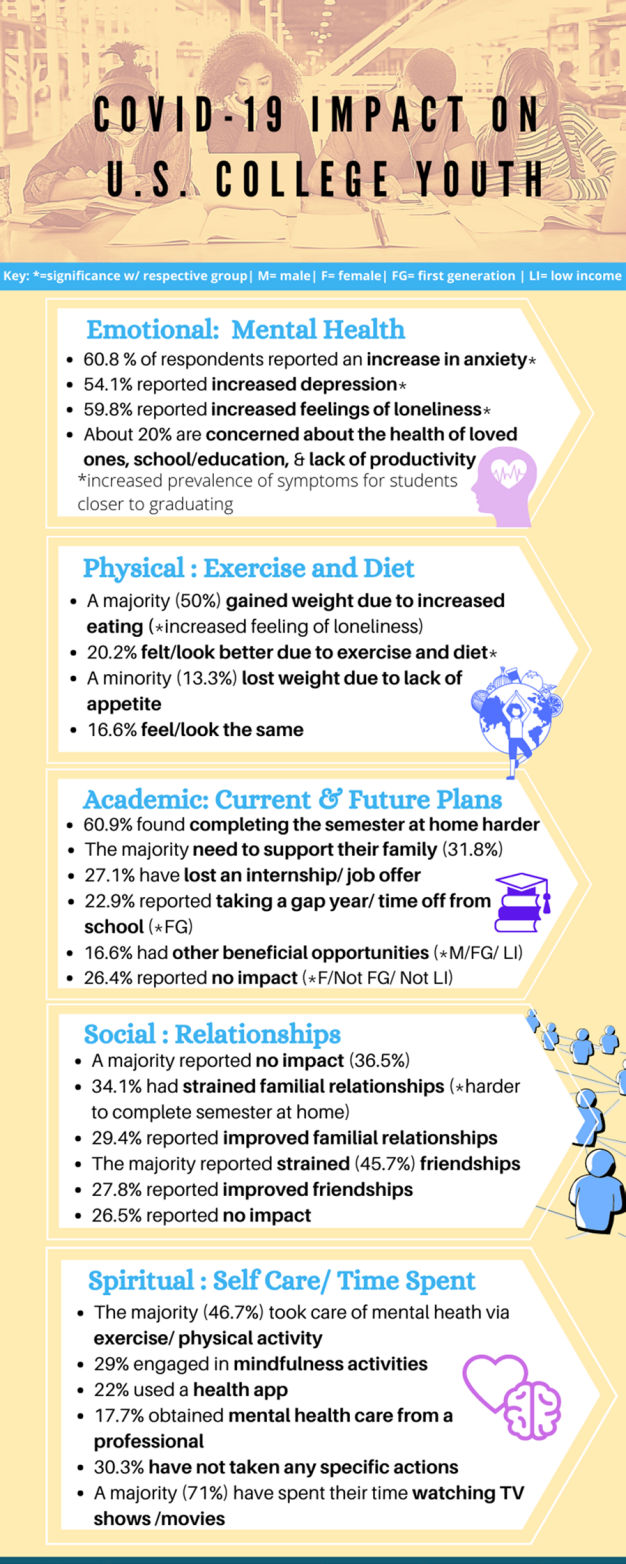
Ways in which COVID-19 has affected the mental health of college students

Based on our survey’s results, the mental health status of the respondents has been most affected by their worries about the health of their loved ones amidst the COVID-19 pandemic. On top of that, many respondents feel uneasy about their plans for their continuing education, anxious about their lack of productivity at home, concerned about their finances and losing job and internship prospects, and worried about contracting the virus. These factors likely made it harder for students to complete the Spring 2020 semester at home, as many respondents have indicated. Indeed, most respondents indicated that their concern about the ongoing pandemic largely began when college campuses began to shut down or when states issued lockdown guidelines shortly after. These events evidently served as a turning point in the mental health of the respondents. As the pandemic ensued, the respondents reported that their lives were disrupted in significant ways. For instance, many reported to have lost an internship or job offer. Others reported the need to take a gap year. In addition, many respondents indicated that they now have the added responsibility of supporting their family in some way. Of the respondents who claimed that the COVID-19 pandemic strained family relationships, most admitted that it has been harder to work from home than at school. These results are summarized in Fig. 4.

More than one third of first-generation students experienced increased anxiety and a quarter found it harder to complete the semester at home. Accordingly, significantly more first-generation students reported taking a gap year or time off from school. First generation students were more likely able to take advantage of opportunities due to the pandemic, indicating access to support systems that may have allowed these students to take time off from school in the first place. Men and low-income students were also more likely to benefit from opportunities due to the pandemic while women, non first-generation, and non low-income students were more likely to report no impacts. However, there were no significant differences across these three demographic statuses, indicating that the main differences were solely in whether or not they gained beneficial opportunities due to the pandemic. Taken together, it appears that first generation and low-income students likely sought out more support during the pandemic, which has helped them overcome pre-pandemic anxiety regarding communicating with campus faculty and staff and utilizing support services [14]. In regards to male students who benefitted from these opportunities, they likely took time off from school as the number of men who have enrolled in college this Fall 2020 declined by seven times compared to the number of women enrolled [15], potentially due to the increased mental health issues experienced by all. The key difference is that men are less likely to seek help for mental health difficulties compared to women even pre-pandemic [16].

On the other hand, there were mixed experiences (albeit statistically insignificant) for LGBTQIA+ students with regard to anxiety, with a quarter reported increased levels and a similar number reporting decreased levels of anxiety. There were similarly mixed experiences in completing the semester from home. This may be due to LGBTQIA+ students accessing necessary resources as sixty-two percent of U.S. colleges and universities have LGBTQIA+ support groups [17] that likely serve as a valuable resource for these students throughout the pandemic.

Presumably due to increased stress factors at home and distance barriers, most respondents indicated that the COVID-19 pandemic strained relationships with their friends. While communications with friends declined, most respondents reported to be spending most of their free time watching TV because of widespread state and local lockdown orders. According to a health survey in England from 2012, watching TV for two hours or more on a daily basis is associated with mental health disorders, measured by a poor performance on the General Health Questionnaire and Warwick-Edinburgh Mental Well-being Scale [18]. On the other hand, nearly 40% of respondents indicated that they spent time exercising. Physical exercise has been proven to alleviate anxiety and depression, while increasing one’s mood and cognitive function [19]. Roughly half of the respondents claimed that they used exercise to take care of their mental health. Of those people, most reported feeling better. However, over 30% of respondents admitted to not specifically taking care of their mental health. Most people reported that they gained weight, particularly those who reported increased loneliness. The isolation and lack of contact may have led affected students to seek comfort food or decrease their daily activity levels, whether it may be training on a sports team or walking to classes and other commitments.

Similar to our findings that the COVID-19 pandemic has led to an overall reduction in the mental health of US college students, a study in China reported that about 40% of female adolescents have suffered from depression. The study cited distance learning, concern about the pandemic, and a decrease in physical exercise and sleep as independent factors causing this increase in depression [20]. Undoubtedly, the pandemic has caused many to experience feelings of loneliness and isolation. Furthermore, it has caused many individuals’ pre-existing mental health conditions to deteriorate without access to the appropriate services [21].

These services are offered on most college campuses but are more difficult to access in the world of online learning. According to a study in the New York City metropolitan area, only about half of the surveyed colleges offered information about remote counseling on their website and less than 60% of college counseling websites even offered directions for students experiencing a mental health emergency [22]. Furthermore, many news outlets report that out-of-state students have been unable to receive mental health services from their schools due to state-dependent licensures—some of which require psychologists to apply for a temporary license or receive special permission to practice [23]. Although some policies have been relaxed, students still face geographic, time, and financial barriers to access these necessary mental health services [23–25]. Additionally, the flexibility extended for telemedicine providers due to the pandemic being a public health emergency began to expire in Fall 2020, and navigating these policies is time-consuming and confusing for healthcare providers [25]. All of these obstacles have contributed to out-of-state students losing their mental health support completely in the face of the clear decline in mental health we have found, as supported by recent similar studies [26–29].

To combat the mental health challenges experienced by many US college students amidst the COVID-19 pandemic, colleges and universities across the country could take steps to expand access to virtual mental health resources and professional guidance. Some institutions have taken to a 24/7 crisis support line as well as virtual resources and emergency funding provided by the CARES Act distributed through the Higher Education Emergency Relief Fund [30]. Some universities were able to refund costs from room and board, which likely contributed to easing financial distress [31]. At the federal level, the PSYPACT agreement was passed—allowing interstate practice within the fourteen states it has been enacted in [32]ーand the TREAT Act that would allow healthcare professionals to render services anywhere during the COVID-19 pandemic was proposed but has yet to be implemented [33].

## Limitations

The survey results are limited by the fact that only students who are registered with Pollfish® as publishers have the opportunity to participate in this survey. Potentially due to the monetary incentive given by Pollfish® to the respondents of their surveys, first generation and low income students are slightly overrepresented. In this survey, 61.0% of students are first generation, 35.5% being low income which is higher than the national average of 56.0% first generation students [34]. However, a study by Haenz et al. at the University of California, Los Angeles reports that roughly 50.0% of first generation students are low-income [35], which may be accounted for by the additional 10.5% of students who preferred not to reveal their income status in our study. Additionally, there are more students who identify as a member of the LGBTQIA+ community (36.5%) compared to the national average of 18.2% [36]. Our slightly disproportionate sample indicates that our findings may not be fully representative of all U.S. college students.

Future avenues of investigation include looking into specific experiences of these groups as well as other factors, such as the experiences of international students, of associate degree program students, community college students, or students completing online degrees and areas of improvement for the specific support systems in place at these educational institutions for these students since the pandemic.

## Conclusion

The outbreak of COVID-19 has taken a universal toll on almost all aspects of life. As cases rapidly increased with great incidence, dense areas and indoor public spaces were closed and physical distancing as well as other preventative measures were enforced. These safety measures led to abrupt closures of schools and educational institutions, and a rushed transition to remote learning. Many reported worries for the health of loved ones most impacted their mental health, and the need to take care of family most affected current and future plans. Given that most students’ concerns were heightened when college campuses sent students home as well as the detrimental effects of the COVID-19 pandemic on students’ mental health, it is crucial for colleges as well as federal regulations to provide the appropriate accommodations and access to mental health care to ensure well-being and safety are prioritized as much asーif not more thanーeducation.

## Supplementary Information

**Additional file 1**. Appendix listing survey questions.

## Data Availability

The datasets used and/or analyzed during the current study available from the corresponding author on reasonable request.

## Abbreviations

CI: Confidence interval
COVID-19: 2019 Novel Coronavirus
LGBTQ: Lesbian, gay, bisexual, transgender, queer or questioning, intersex, and asexual or allied
OR: Odds ratio
TV: Television
LGBTQIA+: Lesbian, Gay, Bisexual, Transgender, Queer, Intersex, Agender, Asexual and other queer-identifying community
PYSPACT: Psychology Interjurisdictional Compact, an interstate compact facilitated by the Association of State and Provincial Psychology Boards (ASPPB)
CARES Act: Coronavirus Aid, Relief, and Economic Security Act, S.3548 bill introduced in 19 March 2020 by Senator McConnell
TREAT Act: Temporary Reciprocity to Ensure Access to Treatment Act, S.4421 bill introduced in 04 August 2020 by Senator Murphy
