# Supplementary material for: Impact of COVID-19 on the mental health of US college students
Source: BMC Psychol. 2021 Jun 8;9:95. doi: 10.1186/s40359-021-00598-3 (PMC8185692; doi:10.1186/s40359-021-00598-3)
Supplement: Supplementary file 1 — Additional file 1. Appendix listing survey questions. [file 40359_2021_598_MOESM1_ESM.docx]

**Impact of COVID-19 on the mental health of US college students**

**APPENDIX: survey instrument**

| Question | Response | Proportion of Respondents |
| --- | --- | --- |
| How has COVID-19 impacted your mental health?  (Multiple responses allowed) | Increased anxiety | 60.8% |
|  | Increased depression | 54.1% |
|  | Increased feeling of loneliness | 59.8% |
|  | Decreased anxiety | 9.1% |
|  | Decreased depression | 5.3% |
|  | Decreased feeling of loneliness | 4.6% |
|  | Other | 1.2% |
|  | N/A - It has not | 10.7% |
| If you are not feeling at ease, what contributes MOST to your mental health status? | Worries for health of loved ones | 20.0% |
|  | Concern about future job offers | 10.8% |
|  | Concern about school/continuing education | 19.2% |
|  | Anxiety about lack of proactivity | 19.0% |
|  | Worries about finances | 15.9% |
|  | Fear of infection | 5.0% |
|  | Other | 3.3% |
|  | N/A - I am feeling at ease | 6.8% |
| Was it easier or harder to complete this semester away from campus? | Easier | 32.7% |
|  | Harder | 60.9% |
|  | No change | 6.4% |
| How has COVID affected your physical health? | I feel/look better due to exercise/dieting | 20.2% |
|  | I gained weight due to increased eating | 50.0% |
|  | I lost weight due to a lack of appetite | 13.3% |
|  | N/A - I feel/look exactly the same | 16.6% |
| How has COVID impacted your current and future plans? (Multiple responses allowed) | Lost an internship or job offer | 27.1% |
|  | Taking a gap year/ time off from school | 22.9% |
|  | Need to support family | 31.8% |
|  | It has given other beneficial opportunities | 16.6% |
|  | Other | 2.5% |
|  | N/A - It has not | 26.4% |
| How has COVID impacted your relationships with your family? | Improved relationships | 29.4% |
|  | Strained relationships | 34.1% |
|  | N/A - No impact on relationships | 36.5% |
| How has COVID impacted your relationships with your friends? | Improved relationships | 27.8% |
|  | Strained relationships | 45.7% |
|  | N/A - No impact on relationships | 26.5% |
| How have you specifically taken care of your mental health amidst COVID-19? (Multiple answers allowed) | Doing mindfulness activities (meditation, yoga, journaling, etc) | 29.0% |
|  | Exercising/engaging in physical activity | 46.7% |
|  | Using a health app | 22.0% |
|  | Obtaining mental health care from a professional | 17.7% |
|  | Other | 1.4% |
|  | N/A - I have not done an activity to specifically take care of my mental health | 30.3% |
| At which point was your concern about COVID-19 heightened? | When the first case of COVID-19 was reported in the US | 19.8% |
|  | When college campuses sent students home | 29.8% |
|  | When states began lockdown guidelines | 29.0% |
|  | When a friend or relative was diagnosed with COVID-19 | 10.5% |
|  | When friend or relatives were taking prevention measures seriously | 6.4% |
|  | Other | 0.3% |
|  | N/A - I am not concerned about COVID-19 | 4.1% |
| How are you spending most of your time during the pandemic?  (Multiple answers allowed) | Watching TV shows and movies | 71.0% |
|  | Reading a book | 30.5% |
|  | Exercising | 39.6% |
|  | Learning new skills or picking up new hobbies | 34.9% |
|  | Cooking/Baking | 33.6% |
|  | Working/Interning | 29.5% |
|  | I'm not really doing anything | 8.0% |
|  | Other | 1.9% |
